# Predictive model of malignancy probability in pulmonary nodules based on multicenter data

**DOI:** 10.3389/fonc.2025.1588147

**Published:** 2025-05-28

**Authors:** Yuyan Huang, Yong Chen, Fang He, Li Jiang

**Affiliations:** Department of Respiratory and Critical Care Medicine, The Affiliated Hospital of North Sichuan Medical College, Nanchong, Sichuan, China

**Keywords:** pulmonary nodules, malignancy, machine learning, prediction model, external test

## Abstract

**Objectives:**

To study the characteristic factors associated with the occurrence of malignant nodules in patients presenting with pulmonary nodules, develop a predictive model, and evaluate its diagnostic performance.

**Methods:**

This study analyzed the clinical and imaging data of 830 patients with pulmonary nodules from the Affiliated Hospital of North Sichuan Medical College. The Least Absolute Shrinkage and Selection Operator (LASSO) and multivariate logistic regression analysis were utilized to identify characteristic predictors. Multiple machine learning classification models were employed for analysis, with the optimal model ultimately selected. A Shapley Additive Explanations (SHAP) framework was developed for personalized risk assessment. Finally, external testing was performed using data from 330 pulmonary nodule patients at Guang’an People’s Hospital.

**Results:**

The predictive factors for malignant pulmonary nodules included: age, gender, nodule diameter, spiculation, lobulation, calcification, vacuole, vascular convergence sign, air bronchogram sign, pleural traction, and density of the nodule. The Gradient Boosting Decision Tree (GBDT) classification model demonstrated optimal performance, with an area under the curve (AUC) of 0.873 (95% confidence interval [CI]: 0.840–0.906) on the internal test set and 0.726 (95% CI: 0.668–0.784) on the external test set. Both the calibration curve and clinical decision curve analysis (DCA) indicated excellent model calibration and substantial clinical benefits.

**Conclusions:**

We developed a GBDT model that provides a basis for differentiating malignant pulmonary nodules, which may assist in the diagnosis and treatment of patients with pulmonary nodules.

## 1 Introduction

A pulmonary nodule refers to a round or oval-shaped, focal, increased-density shadow in the lung observed on imaging, with a diameter of ≤3 cm. With the widespread use of CT scans, the detection rate of pulmonary nodules has continued to rise (1).Pulmonary nodules can be classified into benign and malignant types. Benign nodules are often associated with inflammatory diseases such as tuberculosis and granulomas, while malignant nodules are typically indicative of early-stage lung cancer. Lung cancer remains the most common type of cancer globally, the leading cause of cancer-related deaths, and the disease with the highest global economic burden (2, 3). Prognostic outcomes demonstrate dramatic variation across disease stages, with 5-year survival rates plummeting from 82% in stage IA to merely 7% in stage IVB (4).Early diagnosis and treatment of lung cancer are crucial for improving patient prognosis. Although low-dose spiral CT screening can enhance the detection rate of pulmonary nodules, a significant proportion of nodules initially suspected to be malignant prior to biopsy are ultimately confirmed as benign after pathological examination, which imposes additional clinical risks and financial burdens on patients (5). Therefore, early identification of risk factors and the development of predictive models are of critical significance for improving the early diagnosis, treatment, and prognosis of malignant pulmonary nodules, as well as avoiding unnecessary invasive procedures. Machine learning constitutes a suite of powerful algorithms capable of analyzing, learning from, adapting to, representing, and predicting data. It efficiently addresses multicollinearity among independent variables. Machine learning is widely regarded as the future of computer-aided diagnosis and medical research (6). Therefore, this study collected and organized imaging and clinical data from patients with pulmonary nodules, employed several machine learning classification models to analyze risk factors for malignant nodules, developed a predictive model, and established an evidence-based framework to optimize clinical decision-making in early-stage lung cancer management.

## 2 Materials and methods

### 2.1 Materials

#### 2.1.1 Subjects

**Figure 1:**
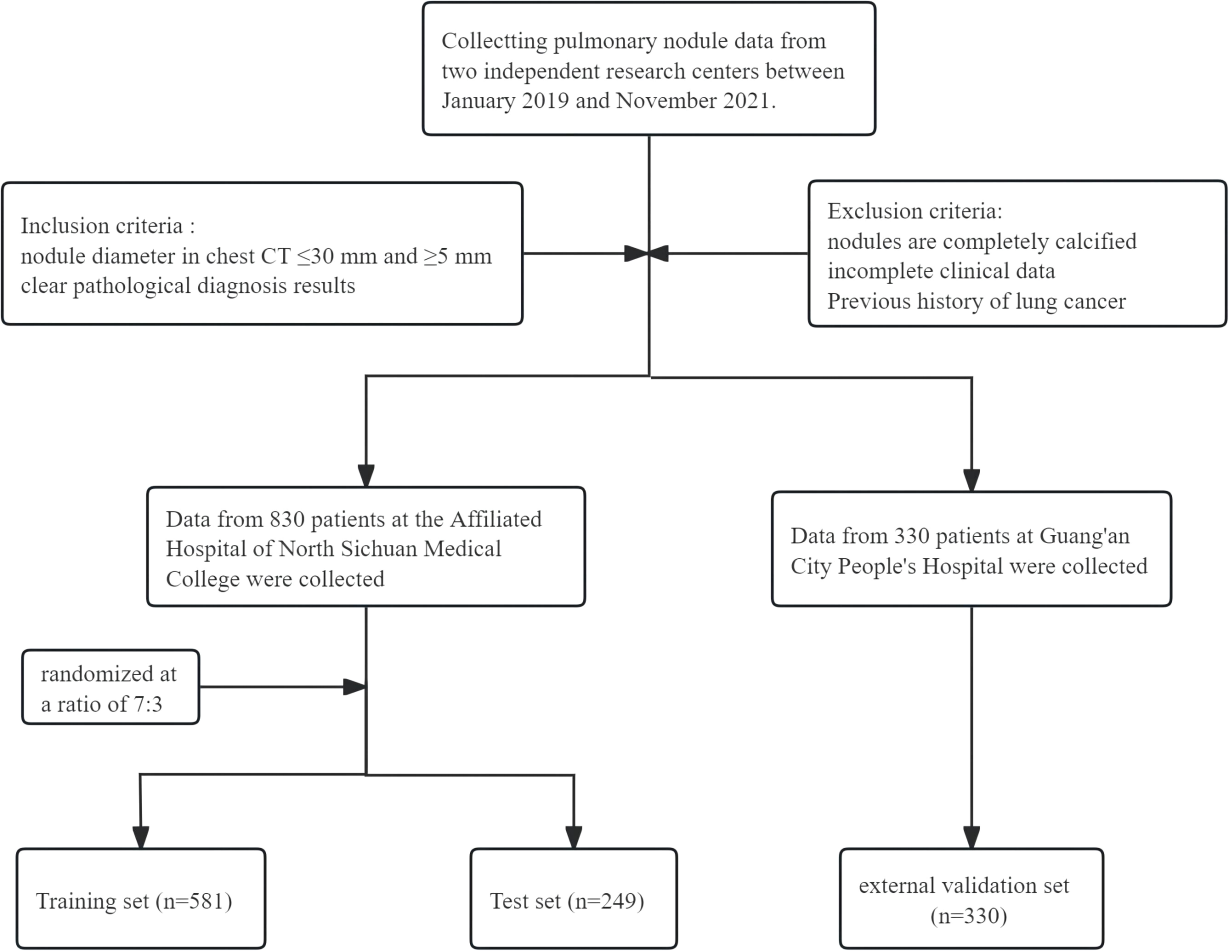
Flowchart of the studied subjects.

A retrospective cohort study was conducted involving 1,160 patients with pulmonary nodules who underwent evaluation at two medical centers: the Affiliated Hospital of North Sichuan Medical College and Guang’an People’s Hospital between January 2019 and November 2021. The patient selection process is shown in **Figure 1**. This study was approved by the Medical Ethics Committee of North Sichuan Medical College Affiliated Hospital (File Number: 2022ER234-1). Since this was a retrospective analysis, the requirement for informed consent from patients was waived.

#### 2.1.2 Inclusion criteria

Inclusion criteria (1): Presence of pulmonary nodules measuring 5–30 mm in diameter on chest CT scan; (2) Availability of definitive pathological diagnosis; (3) Completion of chest CT imaging prior to pathological confirmation.

#### 2.1.3 Exclusion standards

Exclusion criteria: (1) Pulmonary nodules were completely calcified; (2)Patients were with incomplete clinical data; (3) Patients were with previous history of primary lung cancer.

### 2.2 Methods

#### 2.2.1 Study Indicators

There were 23 variables:(1) General Information, including gender, smoking, annual smoking volume, dust exposure history, concomitant disease(Chronic Obstructive Pulmonary Disease, Diffuse Pulmonary Fibrosis, Previous Pulmonary Tuberculosis, Pneumoconiosis), tumor history, family history of tumor, family history of lung cancer, family history of non-lung malignancies, and age. (2) imaging features, including nodule diameter, pleural traction, air bronchogram sign, vascular convergence sign, vacuole, cavity, calcification, shape, lobulation, spiculation, edges (Smooth or rough), location(Left Upper Lobe, Left Lower Lobe, Right Upper Lobe, Right Middle Lobe, Right Lower Lobe), and nodule density (solid/part-solid/pure ground-glass).

#### 2.2.2 Construction and evaluation of predictive models

(a) Screening of characteristic factors: First, least absolute shrinkage and selection operator (LASSO) regression analysis was performed using R software (glmnet 4.1.8) for variable screening and complexity adjustment. Subsequently, the results from LASSO regression analysis were subjected to multivariable logistic regression analysis. Finally, characteristic factors with p < 0.05 were identified. (b) Data splitting: Using Python (version 3.11.4) random module, patients from Affiliated Hospital of North Sichuan Medical College with pulmonary nodules were randomly divided into a training set and a test set in a 7:3 ratio, with 581 cases in the training set and 249 cases in the test set. (c) Analysis of multiple machine learning methods: eXtreme Gradient Boosting (XGBoost), Logistic regression, RandomForest, Gradient Boosting Decision Tree(GBDT), support vector machine (SVM), K-Nearest-Neighbors (KNN) were built by using python (version 3.11.4). Subsequently, we trained and validated the aforementioned parametric models, analyzed the significance of training and validation set metrics across different models, and ultimately selected the optimal model. Python (version 3.11.4) was used to calculate the Area under the Receiver Operating Characteristic (ROC) curve, which is commonly employed to evaluate the discriminative ability of predictive models (7). R software (version 4.2.3) implemented decision curve analysis (DCA) to generate clinical utility plots, thereby enabling the evaluation of both clinical significance and applicability of predictive models (8). Plot a calibration curve using Python to evaluate the model’s predictive capability and assess the consistency between the model’s predicted results and actual outcomes (9). Python was used to plot the precision-recall (PR) curves. PR and the area under the PR curve (AP) can provide complementary information to model evaluation methods (10). (d) Optimal model training, validation, and testing pipeline: Perform 10-fold cross-validation on the training set, and evaluate the model on the test set. Python was used to plot learning curves to assess model fitting and stability for both the training set and validation set (11). (e) We plotted the SHapley Additive exPlanations (SHAP) using Python. SHAP is a method for interpreting the predictions of machine learning models. It is based on the concept of Shapley values, which is an impartial method used in game theory to distribute the benefits of cooperation. The computation of Shapley values takes into account all possible combinations of features and evaluates the marginal contribution of each feature to the output of the model. SHAP can interpret the model’s results and calculate its predictive performance (12). (f) External testing of the model: The cohort of 330 pulmonary nodule patients from Guang’an People’s Hospital served as an external test set. Python (version 3.11.4) was used to plot the ROC curve and a calibration curve. R software (version 4.2.3) was used to construct the DCA.

#### 2.2.3 Statistical analysis

Categorical variables were presented as numbers and percentages and compared using the Chi-square test. Continuous variables were expressed as median and interquartile range (IQR) and compared using the Mann-Whitney U test. Bilateral P-value less than 0.05 indicates statistical significance.

## 3 Results

### 3.1 Baseline data

In this study, we enrolled a total of 1,160 cases, comprising 830 patients from the Affiliated Hospital of North Sichuan Medical College (dataset 1) and 330 patients from Guang’an City People’s Hospital. In Dataset 1, the cohort comprised 388 males (46.7%) and 442 females (53.3%). Among the 243 subjects with benign nodules, 150 were male (61.7%) and 93 were female (38.3%). Of the 587 patients diagnosed with malignant nodules, 238 were male (40.5%) and 349 were female (59.5%). The specific baseline data of the final Dataset 1 is presented in **Table 1**.

**Table 1:** Baseline characteristics in dataset 1.

Variable		All (n=830)	Benignancy (n=243)	Malignancy (n=587)	p
Gender, n (%)	Male	388 (46.7)	150 (61.7)	238 (40.5)	<0.001
	Female	442 (53.3)	93 (38.3)	349 (59.5)	
Smoking, n (%)	No	597 (71.9)	155 (63.8)	442 (75.3)	<0.001
	Yes	233 (28.1)	88 (36.2)	145 (24.7)	
Annual smoking volume, n (%)	<400	598 (72.0)	155 (63.8)	443 (75.5)	<0.001
	>=400	232 (28.0)	88 (36.2)	144 (24.5)	
Dust exposure history, n (%)	No	826 (99.5)	242 (99.6)	584 (99.5)	0.851
	Yes	4 (0.5)	1 (0.4)	3 (0.5)	
Concomitant disease, n (%)	No	770 (92.8)	225 (92.6)	545 (92.9)	0.898
	Yes	60 (7.2)	18 (7.4)	42 (7.2)	
Tumor history, n (%)	No	813 (98.0)	239 (98.4)	574 (97.8)	0.599
	Yes	17 (2.0)	4 (1.6)	13 (2.2)	
Family history of tumor, n (%)	No	818 (98.6)	238 (97.9)	580 (98.8)	0.342
	Yes	12 (1.4)	5 (2.1)	7 (1.2)	
Family history of lung cancer, n (%)	No	820 (98.9)	240 (98.8)	580 (98.8)	0.960
	Yes	10 (1.2)	3 (1.2)	7 (1.2)	
Family history of non-lung malignancies, n (%)	No	825 (99.4)	240 (98.8)	585 (99.7)	0.130
	Yes	5 (0.6)	3 (1.2)	2 (0.3)	
Density of the nodule, n (%)	pure ground-glass	81 (9.8)	10 (4.1)	71 (12.1)	<0.001
	part-solid	208 (25.1)	16 (6.6)	192 (32.7)	
	solid	541 (65.2)	217 (89.3)	324 (55.2)	
Location, n (%)	Right Upper Lobe	266 (32.0)	77 (31.7)	189 (32.2)	0.274
	Right Middle Lobe	69 (8.3)	22 (9.1)	47 (8.0)	
	Right Lower Lobe	170 (20.5)	60 (24.7)	110 (18.7)	
	Left Upper Lobe	190 (22.9)	48 (19.8)	142 (24.2)	
	Left Lower Lobe	135 (16.3)	36 (14.8)	99 (16.9)	
Spiculation, n (%)	No	558 (67.2)	177 (72.8)	381 (64.9)	0.027
	Yes	272 (32.8)	66 (27.2)	206 (35.1)	
Edge, n (%)	Rough	742 (89.4)	207 (85.2)	535 (91.1)	0.011
	Smooth	88 (10.6)	36 (14.8)	52 (8.9)	
Lobulation, n (%)	No	142 (17.1)	49 (20.2)	93 (15.8)	0.132
	Yes	688 (82.9)	194 (79.8)	494 (84.2)	
Shape, n (%)	Irregular	788 (94.9)	224 (92.2)	564 (96.1)	0.020
	Regular	42 (5.1)	19 (7.8)	23 (3.9)	
Calcification, n (%)	No	810 (97.6)	226 (93.0)	584 (99.5)	<0.001
	Yes	20 (2.4)	17 (7.0)	3 (0.5)	
Cavity, n (%)	No	808 (97.3)	239 (98.4)	569 (96.9)	0.246
	Yes	22 (2.7)	4 (1.6)	18 (3.1)	
Vacuole, n (%)	No	726 (87.5)	229 (94.2)	497 (84.7)	<0.001
	Yes	104 (12.5)	14 (5.8)	90 (15.3)	
Vascular convergence, n (%)	No	192 (23.1)	113 (46.5)	79 (13.5)	<0.001
	Yes	638 (76.9)	130 (53.5)	508 (86.5)	
Air bronchogram sign, n (%)	No	634 (76.4)	226 (93.0)	408 (69.5)	<0.001
	Yes	196 (23.6)	17 (7.0)	179 (30.5)	
Pleural traction, n (%)	No	324 (39.0)	110 (45.3)	214 (36.5)	0.018
	Yes	506 (61.0)	133 (54.7)	373 (63.5)	
Age, median[IQR]		57[50.000,66.000]	55[48.000,62.000]	57[51.000,67.000]	<0.001
Nodule diameter, median[IQR]		13[10.000,18.000]	11[8.000,16.000]	13[10.000,19.000]	<0.001

### 3.2 Screening of characteristic factors for lung cancer risk in patients with pulmonary nodules

**Figure 2:**
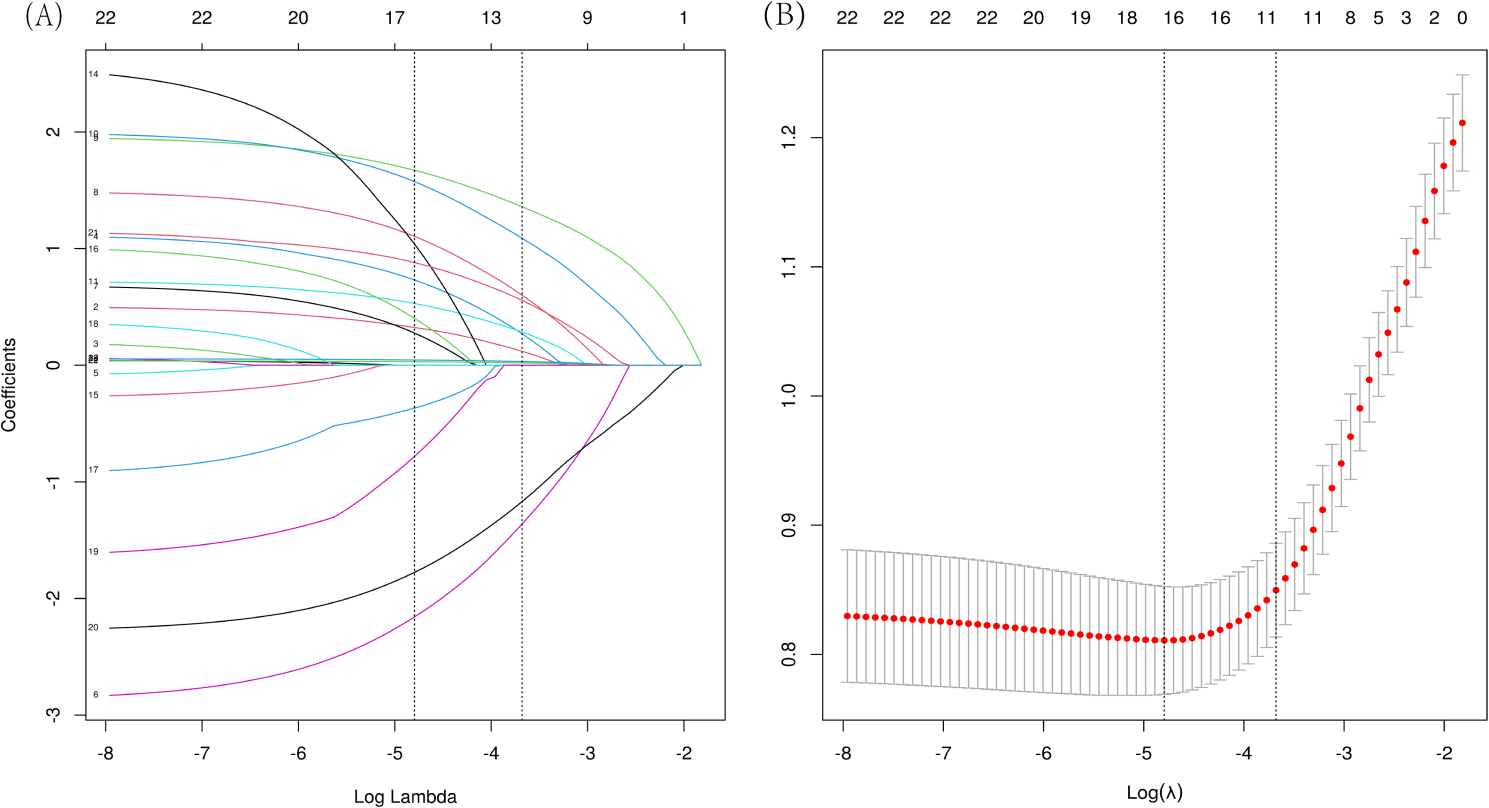
LASSO regression analysis was used to select characteristic factors. **(A)** The use of 10‐fold cross-validation to draw vertical lines at selected values, where the optimal lambda produces eleven nonzero coefficients. **(B)** In the LASSO model, the coefficient profiles of 23 texture features were extracted from logarithmic (λ) sequences. The vertical dashed line is plotted with the minimum mean square error (λ = 0.008) and the error of the minimum distance (λ = 0.025).

Perform LASSO regression analysis on the independent variables, with malignant nodules as the dependent variable (**Figure 2**). The results showed that 16 independent variables were selected (with lambda = 0.008 corresponding to the minimum mean squared error), including spiculation, lobulation, calcification, cavity, vacuole, vascular convergence sign, air bronchogram sign, pleural traction, dust exposure history, tumor history, family history of tumor, family history of non-lung malignancies, density of the nodule, gender, age, and nodule diameter. Then, multivariable logistic regression analysis was used to analyze the aforementioned 16 independent variables. We identified 11 characteristic factors, including age, gender, nodule diameter, spiculation, lobulation, calcification, vacuole, vascular convergence sign, air bronchogram sign, pleural traction, density of the nodule (*p* < 0.05), as **Table 2**.

**Table 2:** Multivariate logistic regression analysis.

Predictor	Estimate	SE	Z	p	Odds Ratio	Lower	Upper
(Intercept)	-3.546	0.709	-5.003	0.0	0.029	0.007	0.115
Age	0.035	0.01	3.522	0.0	1.036	1.016	1.057
Nodule diameter	0.05	0.019	2.605	0.009	1.051	1.013	1.093
Spiculation	0.507	0.237	2.137	0.033	1.66	1.046	2.653
Lobulation	1.105	0.34	3.252	0.001	3.02	1.568	5.968
Calcification	-2.941	0.905	-3.251	0.001	0.053	0.007	0.256
Cavity	0.816	0.681	1.198	0.231	2.261	0.654	9.897
Vacuole	1.604	0.384	4.176	0.0	4.973	2.417	10.968
Vascular convergence	2.056	0.247	8.309	0.0	7.815	4.861	12.851
Bronchiole	2.145	0.328	6.534	0.0	8.541	4.611	16.781
Pleural traction	0.852	0.255	3.343	0.001	2.344	1.43	3.891
Dust exposure history	2.578	1.518	1.699	0.089	13.167	0.766	399.888
Tumor history	1.072	0.852	1.259	0.208	2.922	0.609	17.661
Family history of tumor	-0.459	0.907	-0.505	0.613	0.632	0.112	4.372
Family history of non-lung malignancies	-1.853	1.377	-1.345	0.178	0.157	0.009	2.245
Density of the nodule	-3.168	0.504	-6.29	0.0	0.042	0.015	0.108
Gender	1.038	0.222	4.677	0.0	2.824	1.837	4.391

### 3.3 Analysis of multiple machine learning methods

**Figure 3:**
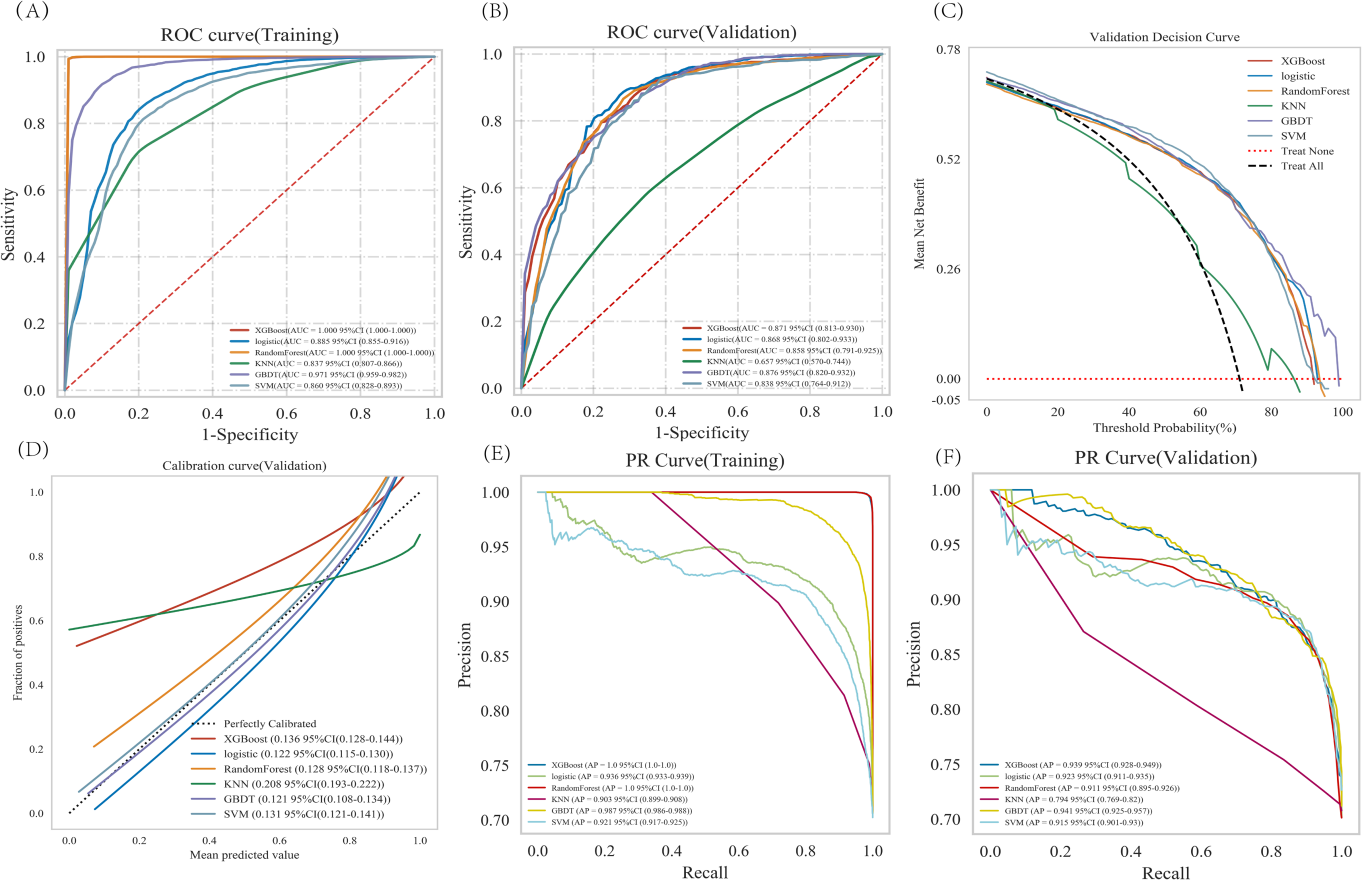
Analysis of multiple machine learning methods. **(A**, **B)** Training set and validation set ROC and AUC. **(C)** In the validation set DCA, the black dashed line indicates the hypothesis that all patients have pulmonary malignant nodules, while the red dashed line represents the alternative hypothesis that none of the patients have malignant nodules. The solid lines correspond to different predictive models. **(D)** For the calibration curve of the validation set, the horizontal axis represents the average predicted probability, the vertical axis denotes the actual probability of the event. The dashed diagonal line serves as a reference, while other smoothed solid lines correspond to the fitting curves of different models. The closer a fitted line is to the reference line (with smaller value in parentheses), the more accurate the model’s prediction is. **(E**, **F)** Training set and validation set PR curve and AP. The y-axis is precision and the x-axis is recall. The higher the AP value, the better the model performance. Different colors in the image represent corresponding models.

XGBoost, Logistic regression, RandomForest, GBDT, SVM, and KNN were trained and repeated 10 times. The evaluation using Area Under the Curve (AUC) values showed that XGBoost and RandomForest achieved the highest scores in the training set, while GBDT attained the highest performance in the validation set (**Figures 3A, B**). The DCA evaluated GBDT with better clinical applicability (**Figure 3C**). The calibration curve indicates better agreement between the predicted probabilities and actual probabilities for both the GBDT and Logistic regression models (**Figure 3D**). The GBDT model demonstrated the best performance in both the training and validation sets, while achieving the highest AP value in the validation set (**Figures 3E, F**). The comprehensive analysis indicated that GBDT could be the optimal model.

### 3.4 Optimal model construction and evaluation

**Figure 4:**
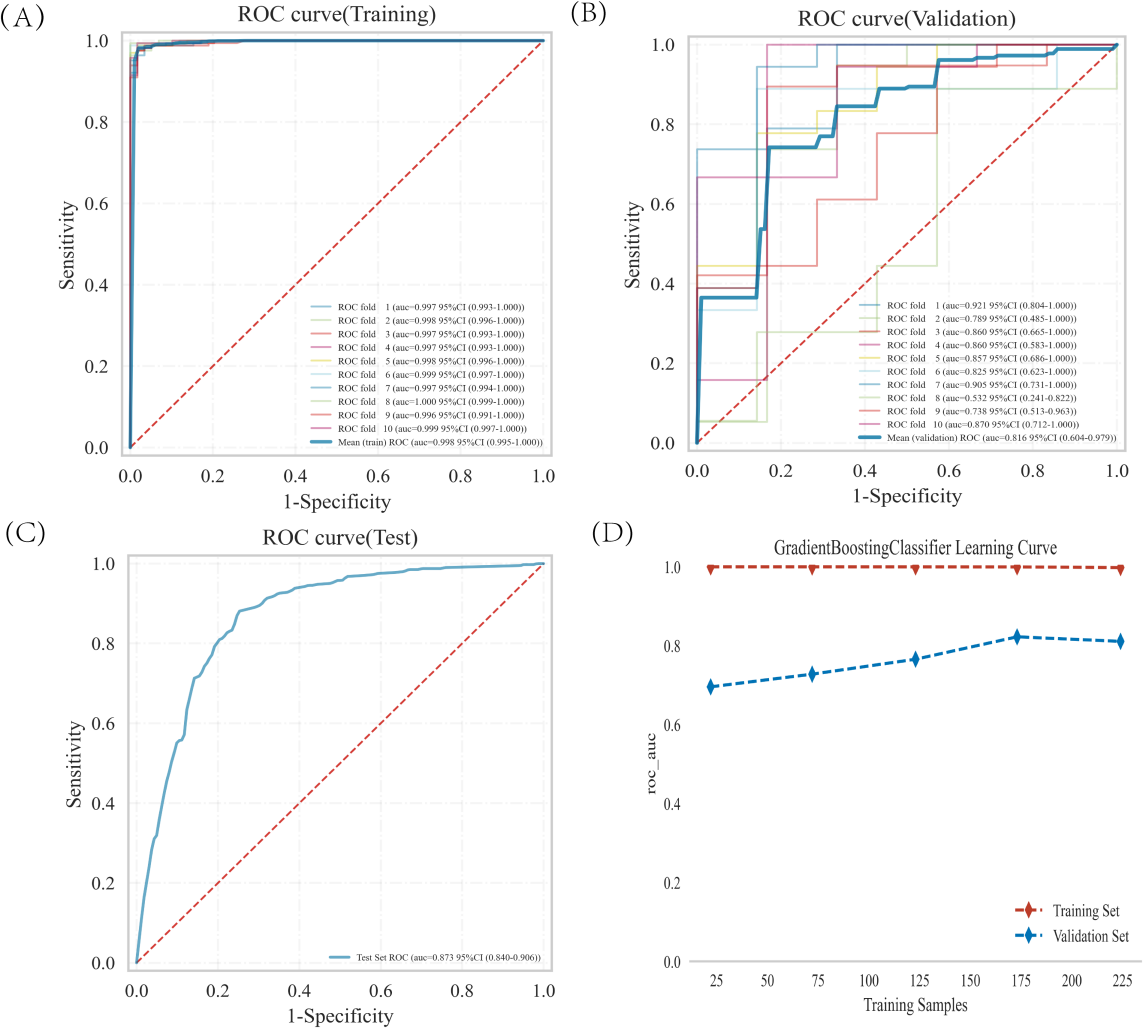
GBDT model training, validation, and testing. **(A**, **B)** Training sets and validation sets ROC and AUC. Different-colored solid lines represent 10 distinct results. **(C)** Test set ROC and AUC. **(D)** Learning curves. The red dashed line represent the training set, and the blue dashed line represent the validation set.

The training set was subjected to GBDT analysis with 10-fold cross-validation. The results show that the validation set had an average AUC of 0.8157 (0.604-0.9789), and the test set achieved an AUC of 0.8727 (0.840-0.906) (**Figures 4A–C**). The model could be deemed successfully fitted as the validation set’s performance under the AUC metric does not exceed that of the test set, or the margin of exceedance was less than 10%. The learning curve indicated that both the training set and validation set demonstrated good stability and fitting (**Figure 4D**). These results indicated that the GBDT model can be applied to our dataset for classification modeling tasks.

### 3.5 Interpretation of the model by SHAP

**Figure 5:**
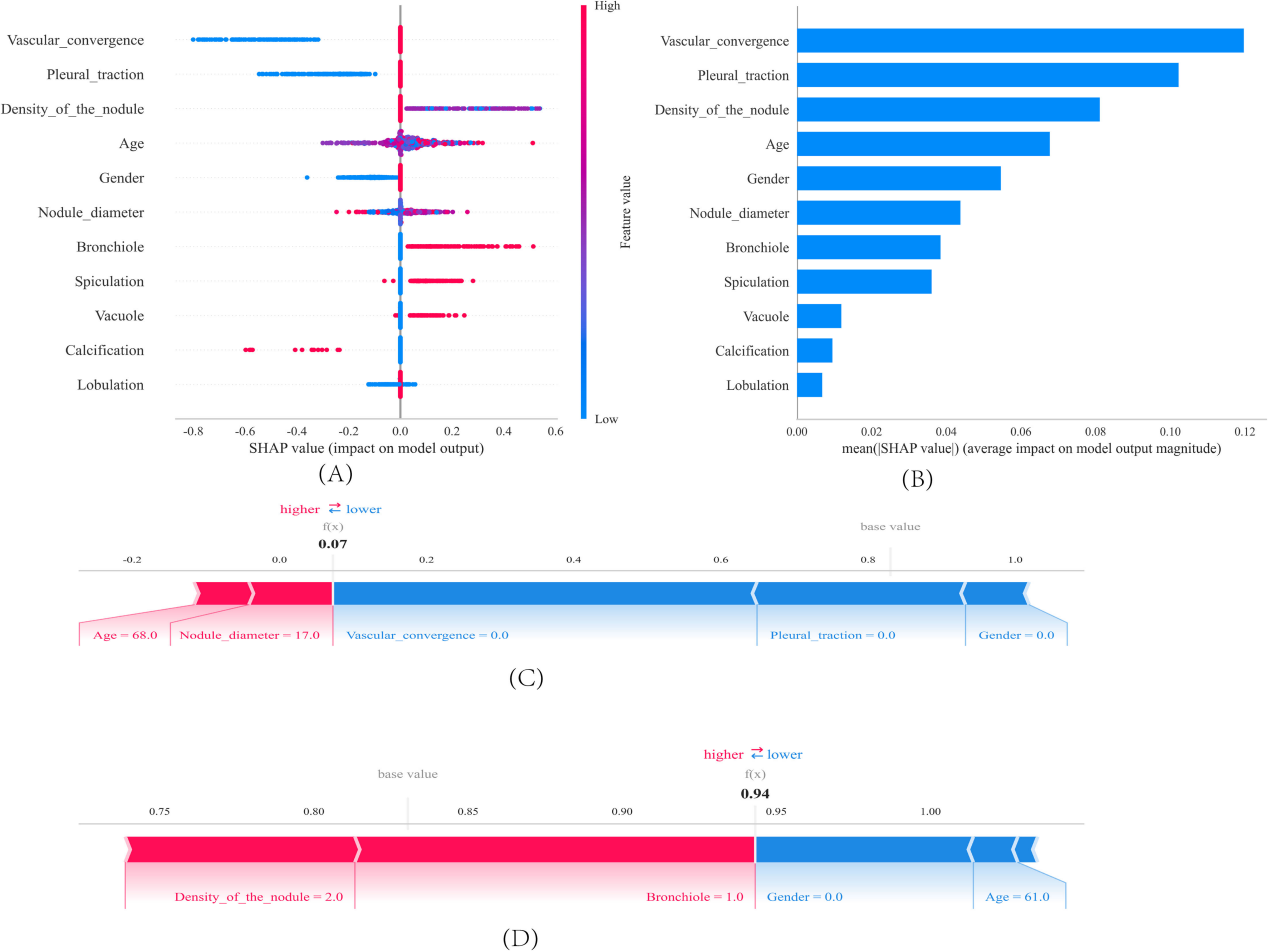
SHAP interprets the model. **(A)** Feature contributions in SHAP. Each line represents a feature, with the x-axis indicating SHAP values. Red dots denote higher feature values, while blue dots indicate lower feature values. **(B)** SHAP-indicated feature importance ranking. The matrix plot illustrates the importance of each covariate in the development of the final predictive model. **(C**, **D)** SHAP Scores in Patients with Benign and Malignant Pulmonary Nodules. SHAP values indicate the contribution of individual patients’ predictive features to the predicted probability. Red features indicate increased risk, while blue features represent reduced risk. The length of the arrows helps visualize the extent to which the prediction is influenced. A longer arrow corresponds to a more significant effect.

**Figure 5A** displays the 11 characteristic factors in our model, which are associated with the malignant risk in patients with pulmonary nodules. Each line corresponding to a feature factor is plotted with dots of different colors; blue dots indicate low risk, while red dots represent high risk. **Figure 5B** shows the ranking of 11 feature factors assessed by the mean absolute SHAP values, where the x-axis SHAP values indicate the importance of the feature factors in the model. We also provide two examples to illustrate the interpretability of the model: one patient with benign pulmonary nodules received a low SHAP prediction score (0.07)(**Figure 5C**), while another patient with malignant pulmonary nodules obtained a significantly higher SHAP score (0.94)(**Figure 5D**).

### 3.6 External testing of the model

**Figure 6:**
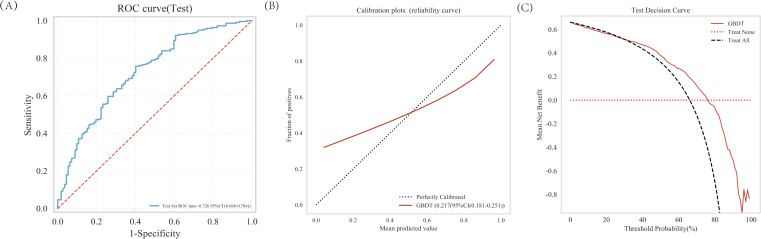
The predictive value and clinical application of the GBDT model in the external test set. **(A)** The ROC curve and AUC of external test set. **(B)** Calibration of the external test set. **(C)** Decision curve analysis of the external test set.

The GBDT analysis conducted on the external test set demonstrated an AUC of 0.726 (**Figure 6A**). The decision curve analysis (DCA) (**Figure 6C**) performed on the external test set demonstrated that implementing interventions within a reasonable range of threshold probabilities might offer greater clinical benefits compared to intervening in all patients or none. The calibration curve of the developed model was evaluated in an external testing cohort, and the results demonstrated a good model fit (**Figure 6B**).

## 4 Discussion

In this study, we included patients with pulmonary nodules measuring 5–30 mm in diameter, and excluded patients with completely calcified nodules. It is very low of the prevalence of malignant pulmonary nodules measuring <5 mm (13). In the NELSON study, malignancy risk in patients with pulmonary nodules measuring <5 mm was similar to subjects without the nodules (14). In addition, the nodule diameter threshold for the need of follow-up has been decided as 5 mm for BTS guidelines (15). So we included nodules of the diameter  ≥5 mm for our research objects. Unlike partially calcified nodule is unclear benign or malignant, completely calcified nodule is benign lesion. Research by Zhou Y et al. (16) showing that 0.90% of partially calcified nodules were diagnosed as malignant and two cases of calcified nodules were benign. Calcification is usually relate to the healing of old lesions and represents stable, benign lesions (17). In particular, diffuse calcifications is highly indicate benign lesions (18).

Our results show that eleven characteristic variables (age, gender, nodule diameter, spiculation, lobulation, calcification, vacuole, vascular convergence, bronchiole, pleural traction, density of the nodule) were screened by LASSO and multivariate logistic regression analysis from 23 clinical and CT variables to assess the risk of lung cancer in patients. These findings align with existing literature on malignant pulmonary nodule assessment. For example, in a large retrospective study, development, and external and internal validation of the model to predict the risk of lung cancer, using data from 19.67 million people has shown that the predictors included age and sex (19).Han DH et al. reported increased age was associated with participants who developed lung cancer (20). A nationwide, prospective cohort, multicenter study have demonstrated that female sex and age older than 60 years were related to an increased risk of invasive lung cancer (21). A recent study reported that the size of pulmonary nodule is the key factor to assess malignancy. The probability of malignant nodules was positively correlated with their diameter (22). Spiculation occurs as tumor cell infiltrate into the adjacent bronchial vascular sheath or local lymphatic vessels, or as tumor-associated fibrous bands stimulate connective tissue formation (23).A new scoring system for predicting malignant pulmonary nodules suggested that spiculation was an independent risk factor. A recent study indicated that features such as spiculation, lobulation had significant predictive value for the malignant nodule (24). Research by Liu et al. (25) supports our finding, showing that lobulation was the Imaging characteristics suggesting malignancy.

The calcification, particularly central and layered calcifications are highly indicative of benign lesions (26, 27). Our study also identified calcification as a protective factor. Vacuoles are areas of low attenuation due to small air within the nodule containing the bronchi. The appearance of vacuole has also been reported to be associated with malignant lung nodules (28). Vascular signs are important indicators of malignant tumors, and tumor growth and metastasis depend on new blood vessels. consistent with previous studies (29), vascular convergence was also identified as independent risk factor for lung nodule in our study. Our study showed that the density of pulmonary nodules is associated with their risk of malignancy. Previous study has demonstrated that, compared to solid nodules, part-solid nodules carry a higher risk of malignancy, while pure ground-glass nodules have the lowest malignant potential (30). Their corresponding malignancy rates were 7%, 63%, and 18%, respectively (31). Pleural traction is typically caused by the traction force exerted on the pleura due to tumor growth around a pulmonary nodule. This traction force may result from tumor cells invading surrounding tissues and extending to the pleural membrane. Bronchiolar signs refers to the presence of lucent shadows resembling bronchial structures within pulmonary nodules on CT scans. This phenomenon is typically caused by either the preservation of partial airway structures within the tumor or the compression of surrounding airways by the tumor. Our findings also indicated that pleural traction and bronchiolar signs were characteristic manifestations of malignant pulmonary nodules, which aligns with previous research findings (32, 33).

In our study, we employed multiple machine learning classification models to construct predictive models. The analysis revealed that the Gradient Boosting Decision Tree (GBDT) model outperformed other models. We applied the SHAP method to the GBDT model, which provided both a more comprehensive interpretation of the predictive model and a more intuitive visualization of prediction outcomes. The results demonstrate that features including lobulation, calcification, vacuole, spiculation, bronchiole, nodule diameter, gender, age, density of the nodule, pleural traction and vascular convergence exhibit a progressively increasing contribution to the model, indicating their gradually enhanced diagnostic value in assessing the malignancy risk of pulmonary nodules. The data for this research were sourced from two independent research centers, and our study incorporated both internal validation, testing and independent external testing components, which has enhanced the generalizability of our research findings.

Our study has several limitations. First, this is a retrospective study, and future prospective studies are needed to further validate its performance. Second, external testing of the model in this research was conducted only at a single medical center; additional data from multiple centers are still required for external testing. Furthermore, due to the extremely low probability of malignancy in nodules smaller than 5 mm in diameter, such cases were not included in our dataset. Future studies are necessary to verify the applicability of our model to nodules with diameters less than 5 mm.

## 5 Conclusions

This study constructed a predictive model based on multiple machine learning classification models, among which the GBDT model demonstrated superior performance. External testing further supported the robustness of our model. We provided personalized risk assessment for early-stage lung cancer development in patients with pulmonary nodules, interpreted through the SHAP method. This computer-aided approach exhibits potential value in the management of pulmonary nodules.

## Data Availability

The original contributions presented in the study are included in the article/supplementary material. Further inquiries can be directed to the corresponding author.
